# ﻿A new species of *Laena* Dejean (Coleoptera, Tenebrionidae) from Sichuan Province, China, with an updated key

**DOI:** 10.3897/zookeys.1221.132774

**Published:** 2024-12-13

**Authors:** Haizhi Wang, Zhonghua Wei, Guodong Ren

**Affiliations:** 1 College of Life Sciences, China West Normal University, 637009, Nanchong, Sichuan Province, China China West Normal University Nanchong China; 2 College of Life Sciences, Hebei University, 071002, Baoding, Hebei Province, China Hebei University Baoding China

**Keywords:** DNA barcoding, Lagriinae, Laenini, new species

## Abstract

In this study, we describe and illustrate a new species of the genus *Laena* Dejean, 1821, *Laenacostata***sp. nov.**, which was collected in Micangshan Nature Reserve of Sichuan Province, China. Additionally, the COI mitochondrial gene was sequenced to provide additional evidence for this new species’ validity. The results of phylogenetic analyses suggest that this new species is sister to *L.maowenica* Schawaller, 2008. Furthermore, an updated key to *Laena* species from Sichuan Province is provided.

## ﻿Introduction

The genus *Laena* Dejean, 1821 belongs to the tribe Laenini Seidlitz, 1895 of the family Tenebrionidae Latreille, 1802 (Bouchard et al. 2021), which is one of the largest genera in the subfamily Lagriinae Latreille, 1825. *Laena* species recorded in China appear endemic, except for *L.leonhardi* Schuster, 1916 and *L.brunkei* Schawaller & Bellersheim, 2023 (Schawaller 2001; Schawaller and Bellersheim 2023), and most *Laena* species appear to have narrow distribution ranges. Given China’s complex terrain and diverse ecological environments, it is likely that many undescribed *Laena* species exist within the country.

The Micangshan Nature Reserve, located in northern part of Sichuan Province, borders on Shaanxi Province. An insect diversity survey was initiated in the Micangshan Nature Reserve from 2023 to 2024, during which the genus *Laena* was found. Specimens of *Laena* were collected by sifting leaf litter. After examining the collected specimens, two *Laena* species were identified: *L.qinlingica* Schawaller, 2001 and *L.costata* sp. nov. This study provided a description and illustrations of this new species, as well as the results of DNA barcoding. Molecular species identification was conducted using newly sequenced COI data (Table 1), along with previously published COI sequences of *Laena* species (Wei and Ren 2023, 2024). An updated key to *Laena* species from Sichuan Province, modified from Wei et al. (2020), is provided. Seven *Laena* species (*L.baogua* Schawaller, 2021, *L.chunyang* Schawaller, 2021, *L.dentithoraxa* Wei & Ren, 2023, *L.grebennikovi* Schawaller, 2021, *L.mounigouica* Wei & Ren, 2023, *L.wannian* Schawaller, 2021, and *L.costata* sp. nov.) are added to this key.

**Table 1.** COI GenBank accession numbers and voucher information of *Laena* species provided in this study.

Name	Collection site	GenBank accession no.
*Laenaqinlingica* Schawaller, 2001	China, Sichuan Province, Wangcang County, Micangshan Nature Reserve, Jinchangba, 32.4985°N, 106.6234°E, elev. 1880 m	PQ059651
*Laenacostata* sp. nov.	China, Sichuan Province, Wangcang County, Micangshan Nature Reserve, Jinchangba, 32.4985°N, 106.6234°E, elev. 1880 m	PQ059650
*Laenahabashanica* Schawaller, 2001	China, Yunnan Province, Habaxueshan, Habacun, elev. 2870 m	PQ059648
*Laenatryznai* Schawaller, 2001	China, Sichuan Province, Xiangcheng County, Redazhen, elev. 3200 m	PQ059647
*Laenaquinquagesima* Schawaller, 2008	China, Yunan Province, Xianggelila, elev. 3200–3500 m	PQ059649

## ﻿Materials and methods

The examined specimens of the genus *Laena* were collected in the Micangshan Nature Reserve of Sichuan Province, China, and are deposited at the Museum of China West Normal University (MCWNU). All examined specimens were collected by sifting leaf litter. The specimens were examined using an Olympus SZX10 stereomicroscope. Images were taken using a Canon EOS 9D Mark III camera with a Laowa FF 25 mm F2.8 Ultra Macro 2.5–5× lens.

The sequences of the mitochondrial gene COI were used for molecular species identification. The leg muscles were used for DNA extraction using the Ezup Column Animal Genomic DNA Purifcation Kit (Shanghai, China). The sequences were obtained using polymerase chain reaction (PCR) amplification with the primer pair LCO1490 and HCO2198 (Folmer et al 1994) and the settings used in Wei and Ren (2023). The PCR products were sequenced by Sangon Biotech Co. Ltd (Shanghai, China) after examined using 1.0% agarose gel electrophoretic analysis.

*Anaedusbrunneus* (Ziegler, 1844), *Grabulaxdarlingtonia* Kanda, 2016, and three species of the genus *Hypolaenopsis* Masumoto, 2001 were chosen as outgroups in this study. The newly available DNA sequences were checked and edited using SeqMan v. 7.1.0. Then the new COI sequences (Table 1) and previously known 34 sequences (GenBank accession nos. OR682144–OR682149, OR721926, OR721927, OR721930–OR721939, OR721941–OR721953) were aligned using Clustal W (Thompson et al. 1994) and trimmed using trimmAl v. 1.2 (Capella-Gutiérrez et al. 2009). Based on Bayesian information criterion, the best substitution model, GTR+I+G4+F, was calculated using ModelFinder (Kalyaanamoorthy et al. 2017) that plugged into PhyloSuite v. 1.2.2 (Zhang et al. 2020). The maximum-likelihood (ML) tree was constructed using IQ-TREE v. 1.6.6 (Nguyen et al. 2015) which was also integrated in PhyloSuite. To estimate node reliability, we performed ML analysis using 1,000 ultrafast bootstrapping and 1,000 SH-aLRT iterations.

## ﻿Results

### ﻿Tribe Laenini Seidlitz, 1895

**Genus *Laena* Dejean, 1821**

#### ﻿ Laena costata sp. nov.

Taxon classificationAnimaliaColeopteraTenebrionidae

CF9610CC-B684-5231-8D12-48DC736AFF21

https://zoobank.org/FF55FC90-0D17-4216-8A80-68263966C07A

Fig. 1A–F

##### Type material.

***Holotype***: China • ♂; Sichuan Province, Wangcang County, Micangshan Nature Reserve, Jinchangba; 32.4985°N, 106.6234°E, elev. 1880 m; 2024-IV-21; Zhonghua Wei leg.; MCWNU. ***Paratypes***: China – Sichuan Province • 1♀; Wangcang County, Micangshan Nature Reserve, Jinchangba; 32.4985°N, 106.6234°E, elev. 1880 m; 2024-IV-21; Zhonghua Wei leg.; MCWNU • 1♂; Wangcang County, Micangshan Nature reserve, Shiziba; 32.6554°N, 106.5581°E, elev. 1750 m; 2023-IX-7; Zhonghua Wei leg.; MCWNU.

##### Description.

**Figure 1.**
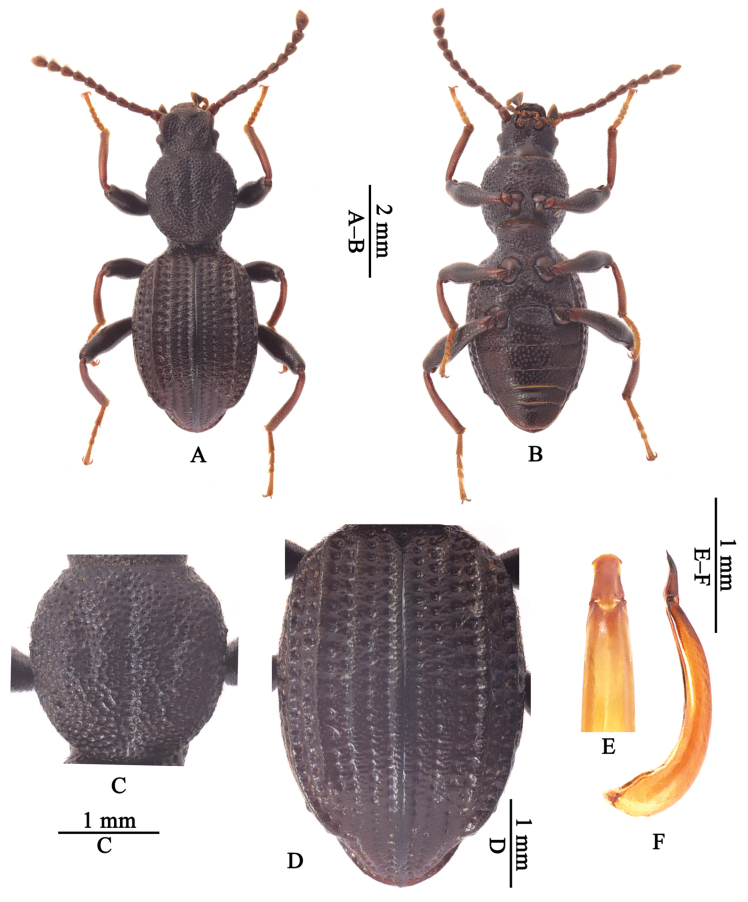
The holotype of *Laenacostata* sp. nov. **A, B** habitus, in dorsal and ventral views **C** pronotum **D** elytra **E, F** aedeagus, in dorsal (apical portion) and lateral views.

**Male.** Body length 6.4–7.2 mm. Body (Fig. 1B) blackish brown, antennae, maxillary palps, and tibiae reddish brown, tarsi light brown; body dorsum rough and covered with dense punctures bearing short setae. Epistome trapezoidal, each lateral part with two longer setae, surface with dense large punctures bearing short setae; anterior margin distinctly concave. Fronto-clypeal suture shallow, straight at middle. Genae ridge-like, strongly raised; surface with dense small punctures. Eyes elliptical and slightly prominent laterally. Frons slightly prominent at middle of anterior portion; surface with dense and large punctures, each puncture with a short seta. Antennae reaching posterior margin of pronotum; antennomere III about 1.7× as long as antennomere II.

Pronotum (Fig. 1C) nearly circular, widest at anterior 1/3, approximately as wide as long; anterior margin nearly straight; lateral margins finely beaded; posterior margin neither bent downwards nor beaded; disc slightly convex, with a longitudinal groove and a pair of shallow depressions at middle, surface with dense and large punctures, distance between punctures equal to 0–1× puncture diameter, each puncture with a short seta; anterior angles rounded and posterior angles obtuse. Prothoracic hypomera with punctures as large as those on pronotal disc, bearing short setae. Prosternal process widest at posterior margin, bent downwards behind coxae; surface with fused large punctures which bear very short setae.

Elytra (Fig. 1D) elongate-oval, approximately 1.5 times as long as wide, widest at middle; strongly prolonged at apices; humeral angles rounded; lateral sides curved; surface rough, without striate, with rows of punctures; punctures in rows bearing short setae, distinctly larger than those on pronotum; intervals with dense fine punctures which bear short setae, intervals I, II, IV and VI flat, III slightly convex, V and VII distinctly convex and ridged, IX with two setigerous pores at posterior portion.

Legs slender. Femora without teeth on inner sides. Tibiae hooked at inner apex; mesotibiae slightly curved on inner sides.

Punctures on abdominal ventrites gradually becoming smaller from ventrite I to V.

Aedeagus (Fig. 1E, F) subfusiform, 1.94–1.96 mm in length. Parameres trapezoidal, widest at base and gradually narrowed from base to apex; apex abruptly widened and strongly arcuated.

**Female.** All the tibiae not hooked at inner sides of apex.

##### Diagnosis.

In the phylogenetic tree (Fig. 3), *Laenacostata* sp. nov., *L.maowenica* Schawaller, 2008, and *L.bifoveolata* Reitter, 1889 form a clade, and the new species appear sister to *maowenica* Schawaller, 2008, but the relationships are not statistically supported. Based on morphological characteristics, *Laenacostata* sp. nov. is similar to *L.bifoveolata* Reitter, 1889, *L.bowaica* Schawaller, 2001, *L.haigouica* Schawaller, 2001, *L.maowenica* Schawaller, 2008 and *L.mounigouica* Wei & Ren, 2023 shared with them body surface having the dense punctures, the pronotal disc with a longitudinal groove and two median impressions, and non-dentate femora without teeth. This new species can be distinguished from the latters by the following characters: (1) body surface rough, with dense punctures; (2) elytral interval III slightly convex, intervals V and VII distinctly convex and ridged; (3) parameres with apex distinctly broadened and constricted at sides near apex; (4) humeral angles arcuated and not prominent; (5) male tibiae hooked at inner apex.

##### Distribution.

China: Sichuan.

##### Etymology.

The name is in reference to the elevated and ridged elytral intervals V and VII; *costata*, Latin, meaning ribbed.

#### ﻿ Laena qinlingica

Taxon classificationAnimaliaColeopteraTenebrionidae

Schawaller, 2001

431256EB-783C-5049-993A-3E0491A3D7CB

Fig. 2A, B

Laena qinlingica Schawaller, 2001: 32; Schawaller 2008: 406; Yuan and Ren 2018: 700; Wei et al. 2020: 527.

##### Examined material.

China – Sichuan Province • 2♀ (in 95% ethanol); Wangcang County, Micangshan Nature reserve, Shuiliandong; 2023-IX-8; Zhonghua Wei leg.; MCWNU • 2♂5♀ (2♂4♀ in 95% ethanol); Wangcang County, Micangshan Nature reserve, Jinchangba; 32.4985°N, 106.6234°N, elev. 1880 m; 2024-IV-21; Zhonghua Wei leg.; MCWNU.

##### Distribution.

China: Sichuan, Shaanxi.

### ﻿An updated key to *Laena* species from Sichuan Province modified from Wei et al. (2020)

The couplets 5, 10, 11, 25 and 41 of the key to *Laena* from Sichuan Province provided by Wei et al. (2020) should be modified as follows to include *Laenabaogua* Schawaller, 2021, *L.chunyang* Schawaller, 2021, *L.costata* sp. nov., *L.dentithoraxa* Wei & Ren, 2023, *L.grebennikovi* Schawaller, 2021, *L.mounigouica* Wei & Ren, 2023, and *L.wannian* Schawaller, 2021.

**Figure 2.**
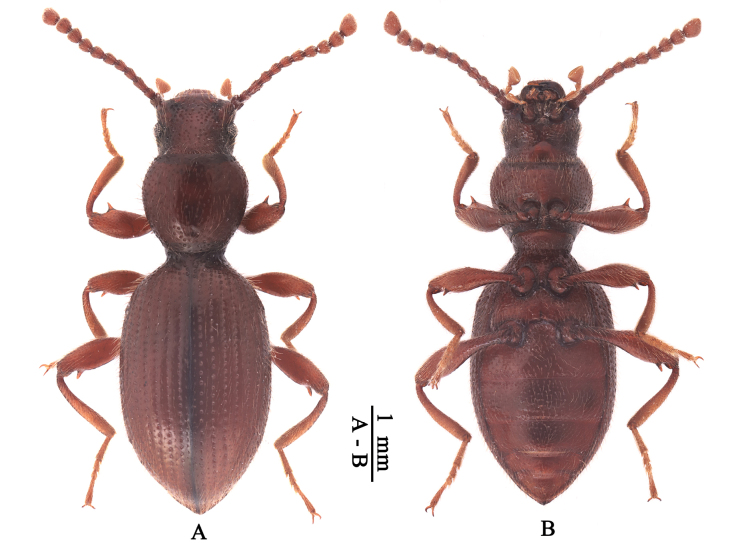
The habitus of *Laenaqinlingcia* Schawaller, 2001 **A** habitus in dorsal view **B** habitus in ventral view.

**Figure 3.**
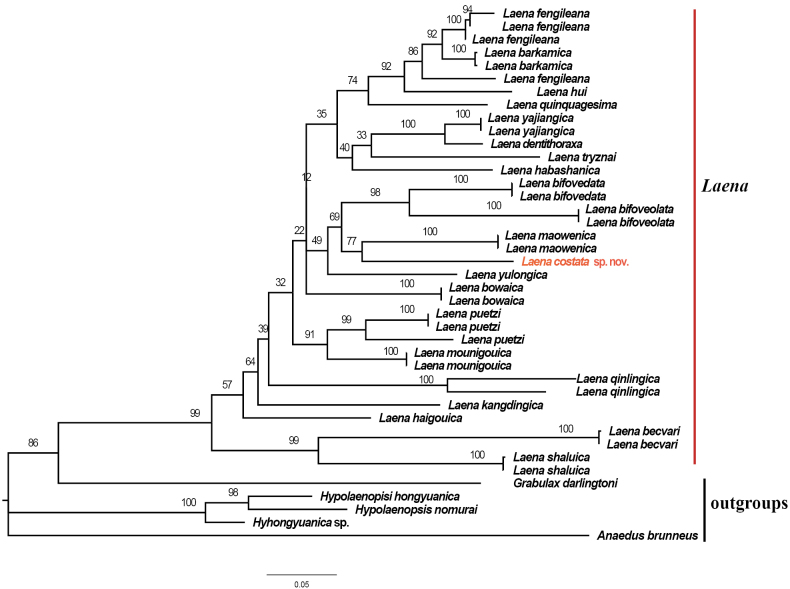
The maximum-likelihood tree of *Laena* species based on available COI sequences. The values on the branches show ultrafast bootstrap supports.

**Table:** 

5	Femora with opposite teeth	**5a**
–	Femora each with a tooth or angle or spine	**6**
5a	Lateral margins of pronotum beaded; pro- and mesofemora each with single tooth, metafemora with opposite teeth	***L.grebennikovi***
–	Lateral margins of pronotum not beaded; all femora with opposite teeth	**5b**
5b	Male protibiae strongly curved at basal third; pronotum with feeble longitudinal impression and on each side with an indistinct impression; body length 8.2–8.5 mm	***L.baogua***
–	Male protibiae weakly curved at base; pronotum with an impression at middle of base; body length 10.6–11.9 mm	***L.latitarsia***
…
10	Pronotum with lateral margins not beaded	**10a**
–	Pronotum with lateral margins beaded or partially beaded	**16**
10a	Lateral margins of pronotum serrated	***L.dentithoraxa***
–	Lateral margins of pronotum arcuated	**11**
11	Elytral intervals V and VII convex or only interval VII convex; male tibiae not hooked or meso- and metatibiae hooked at inner apex	**11a**
–	Elytral intervals V and VII not convex; male metatibiae hooked at inner apex	**13**
11a	Male tibiae not hooked at inner apex	**11b**
–	Male meso- and metatibiae hooked at inner apex	**12**
11b	Pronotal disc with a pair of impressions at middle; elytral intervals with a few scattered fine punctures	***L.chunyang***
–	Pronotal disc without impressions at middle; elytral intervals with a row of fine punctures	***L.wannian***
…
25	Elytral interval VII convex, swollen and knob-shaped at shoulder	**25a**
–	Elytral interval VII not convex and not knob-shaped at shoulder	**27**
25a	Lateral margins of pronotum beaded; elytral interval VII not swollen	***L.costata* sp. nov.**
–	Lateral margins of pronotum not beaded; elytral interval VII swollen	**26**
…
41	Elytral intervals with small scattered punctures; all male tibiae hooked at inner apex	**41a**
–	Elytral intervals with a regular row of small punctures; male meso- and metatibiae hooked at inner apex, without granules	**42**
41a	Male metatibiae with granules on inner sides; elytral intervals with a row of punctures, interval IX with two setiferous pores	***L.hengduanica***
–	Male metatibiae without granules on inner sides; elytral intervals with scattered fine punctures, interval IX with three setiferous pores	***L.mounigouica***

## Supplementary Material

XML Treatment for Laena costata

XML Treatment for Laena qinlingica

## ﻿Additional information

### Author ORCIDs

Zhonghua Wei https://orcid.org/0000-0001-7349-9939

Guodong Ren https://orcid.org/0000-0001-5808-9122

### Data availability

All of the data that support the findings of this study are available in the main text.
